# Supplementary material for: Information bounds on the accuracy of cell polarization
Source: PLoS One. 2025 Sep 30;20(9):e0333522. doi: 10.1371/journal.pone.0333522 (PMC12483228; doi:10.1371/journal.pone.0333522)
Supplement: S1 Fig — (PDF) [file pone.0333522.s001.pdf]

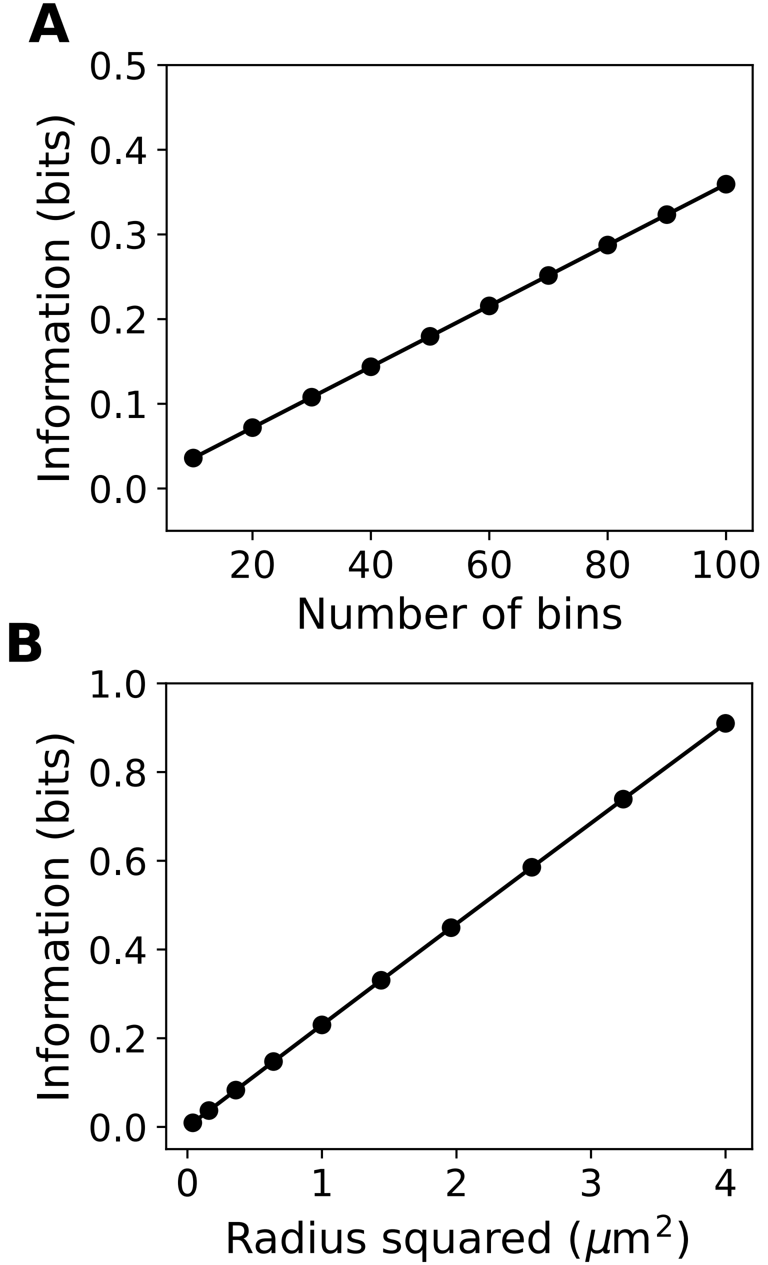

S1 Fig. Relationship between information and number of bins or radius. The total information was calculated using the Gaussian channel approximation (Eq 3) with a signal-to-noise ratio of $g/N^{0.5}$ = 0.1. **A:** Relationship between information and number of bins. The theoretical maximum information from a gradient is shown as a function of the number of bins (*r* = 1). There is a linear relationship as described in the signal-to-noise perspective section in the main text. **B:** Relationship between information and radius squared. The theoretical maximum information from a gradient is shown as a function of the radius squared (n_b = 64).
