# Supplementary material for: Information bounds on the accuracy of cell polarization
Source: PLoS One. 2025 Sep 30;20(9):e0333522. doi: 10.1371/journal.pone.0333522 (PMC12483228; doi:10.1371/journal.pone.0333522)
Supplement: S2 Fig — (PDF) [file pone.0333522.s002.pdf]

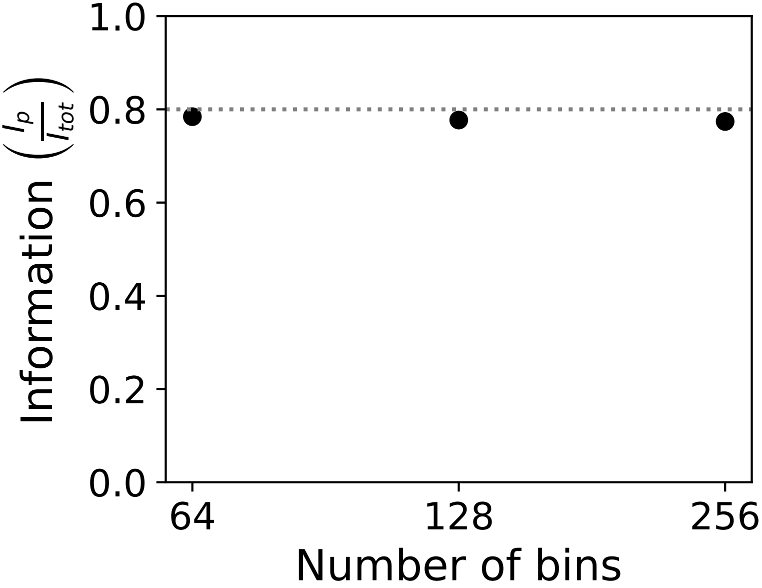

S2 Fig. Calculation of polarization information on three-dimensional sphere. The polarization information (I_p) was computed for a spherical cell containing n_b = 64, 128, 256 bins subjected to a gradient of slope g = 0.01 corrupted by noise N = 1 as described in the Methods section. It was compared to the total information, which was determined using the Gaussian channel approximation. The data are plotted as the ratio of polarization information to total information with the dotted line indicating a ratio of 0.8.
