# Supplementary material for: Information bounds on the accuracy of cell polarization
Source: PLoS One. 2025 Sep 30;20(9):e0333522. doi: 10.1371/journal.pone.0333522 (PMC12483228; doi:10.1371/journal.pone.0333522)
Supplement: S1 Table — (PDF) [file pone.0333522.s003.pdf]

S1 Table. Data for Fig 3B and Fig 3C.

**Fig 3B**		
$g/\sqrt{N}$	I_p/I_{tot}	SD
1	0.290	9.5×10^{-6}
0.5	0.525	1.0×10^{-5}
0.2	0.726	9.5×10^{-5}
0.1	0.768	1.7×10^{-4}
0.05	0.779	1.1×10^{-4}
0.02	0.782	8.4×10^{-4}
0.01	0.783	1.5×10^{-3}
0.005	0.781	1.0×10^{-3}
0.002	0.785	1.4×10^{-3}
0.001	0.782	5.0×10^{-3}

**Fig 3C**		
n_b	I_p/I_{tot}	SD
4	0.637	3.2×10^{-3}
8	0.748	1.5×10^{-3}
16	0.776	2.5×10^{-3}
32	0.783	1.5×10^{-3}
64	0.784	4.3×10^{-4}
128	0.785	3.7×10^{-4}

Mean (I_p/I_{tot}) and standard deviation (SD) for 3 trials.
