# Supplementary material for: Information bounds on the accuracy of cell polarization
Source: PLoS One. 2025 Sep 30;20(9):e0333522. doi: 10.1371/journal.pone.0333522 (PMC12483228; doi:10.1371/journal.pone.0333522)
Supplement: S2 Table — (PDF) [file pone.0333522.s004.pdf]

S2 Table. Data for Fig 4B.

	**Coop**		**PF**		**FA**	
$\sqrt{N}$	I_p	SD	I_p	SD	I_p	SD
0.01	0.19	3.2×10^{-5}	1.1	5.3×10^{-3}	1.2	8.4×10^{-4}
0.02	0.055	3.2×10^{-5}	0.92	1.9×10^{-2}	1.2	1.2×10^{-3}
0.05	0.0091	2.3×10^{-5}	0.46	9.2×10^{-3}	1.2	1.6×10^{-3}
0.1	0.0023	2.2×10^{-5}	0.26	9.4×10^{-4}	1.1	8.4×10^{-3}
0.2	0.00058	6.0×10^{-6}	0.10	1.1×10^{-3}	0.94	1.3×10^{-2}
0.5	0.00010	4.2×10^{-6}	0.01	5.2×10^{-4}	0.54	2.7×10^{-3}

Mean (I_p) and standard deviation (SD) for 3 trials.
