# Supplementary material for: Information bounds on the accuracy of cell polarization
Source: PLoS One. 2025 Sep 30;20(9):e0333522. doi: 10.1371/journal.pone.0333522 (PMC12483228; doi:10.1371/journal.pone.0333522)
Supplement: S3 Table — (PDF) [file pone.0333522.s005.pdf]

S3 Table. Parameters for noise calculation.

Parameter	Description	Default	Bounds	Reference
r	cell radius	2 μm	2 - 4 μm	[1]
n_b	receptors per cell	10,000	5000 - 20,000	[2, 3]
D	α-factor diffusion constant	100 $\mu\mathrm{m}^2\mathrm{s}^{-1}$	$\sim$ 100 $\mu\mathrm{m}^2\mathrm{s}^{-1}$	[4, 5]
s	receptor neighborhood radius	0.01 μm	0.004 - 0.04 μm	*
c_0	mean α-factor concentration	20 nM	20 nM	[6]
K_d	receptor dissociation constant	5 nM	5 - 10 nM	[2, 3]
k_a	receptor-ligand association rate	2×10^6 $\mathrm{M}^{-1}\mathrm{s}^{-1}$	4×10^3 - 2×10^6	S4 Table
T	integration time	10000 s	1000 – 10000 s	[6, 7]

*Range spans receptor diameter to separation of receptors on cell surface.

References

[1] Sherman F. Getting started with yeast. In: Guthrie C, Fink GR, editors. Methods in Enzymology. vol. 350 of Guide to Yeast Genetics and Molecular and Cell Biology - Part B. Academic Press; 2002. p. 3–41. Available from: `https://www.sciencedirect.com/science/article/pii/S007668790250954X`.

[2] Jenness DD, Burkholder AC, Hartwell LH. Binding of alpha-factor pheromone to Saccharomyces cerevisiae a cells: dissociation constant and number of binding sites. Mol Cell Biol. 1986;6(1):318–320.

[3] Yi TM, Kitano H, Simon MI. A quantitative characterization of the yeast heterotrimeric G protein cycle. Proc Natl Acad Sci U S A. 2003;100(19):10764–10769. doi:10.1073/pnas.1834247100.

[4] Young ME, Carroad PA, Bell RL. Estimation of diffusion coefficients of proteins. Biotechnology and Bioengineering. 1980;22(5):947–955. doi:10.1002/bit.260220504.

[5] Chen W, Nie Q, Yi TM, Chou CS. Modelling of Yeast Mating Reveals Robustness Strategies for Cell-Cell Interactions. PLOS Computational Biology. 2016;12(7):e1004988. doi:10.1371/journal.pcbi.1004988.

[6] Chou CS, Bardwell L, Nie Q, Yi TM. Noise filtering tradeoffs in spatial gradient sensing and cell polarization response. BMC Systems Biology. 2011;5(1):196. doi:10.1186/1752-0509-5-196.

[7] Pringle JR, Bi E, Harkins HA, Zahner JE, Virgilio CD, Chant J, et al. Establishment of Cell Polarity in Yeast. Cold Spring Harb Symp Quant Biol. 1995;60:729–744. doi:10.1101/SQB.1995.060.01.079.
