# Supplementary material for: Information bounds on the accuracy of cell polarization
Source: PLoS One. 2025 Sep 30;20(9):e0333522. doi: 10.1371/journal.pone.0333522 (PMC12483228; doi:10.1371/journal.pone.0333522)
Supplement: S4 Table — (PDF) [file pone.0333522.s006.pdf]

S4 Table. Experimental measurements of alpha-factor receptor association rate constant.

Date [Reference]	k_a ($M^{-1}s^{-1}$)
1983 [1]	3×10^3
1986 [2]	3×10^5
1988 [3]	7×10^4
2003 [4]	2×10^6
2004 [5]	$2 - 5 \times 10^5$
2014 [6]	4×10^3

References

[1] Jenness DD, Burkholder AC, Hartwell LH. Binding of α-factor pheromone to yeast a cells: Chemical and genetic evidence for an α-factor receptor. Cell. 1983;35(2):521–529. doi:10.1016/0092-8674(83)90186-1.

[2] Jenness DD, Burkholder AC, Hartwell LH. Binding of alpha-factor pheromone to Saccharomyces cerevisiae a cells: dissociation constant and number of binding sites. Mol Cell Biol. 1986;6(1):318–320.

[3] Raths SK, Naider F, Becker JM. Peptide analogues compete with the binding of alpha-factor to its receptor in Saccharomyces cerevisiae. J Biol Chem. 1988;263(33):17333–17341.

[4] Yi TM, Kitano H, Simon MI. A quantitative characterization of the yeast heterotrimeric G protein cycle. Proc Natl Acad Sci U S A. 2003;100(19):10764–10769. doi:10.1073/pnas.1834247100.

[5] Bajaj A, Ćelić A, Ding FX, Naider F, Becker JM, Dumont ME. A Fluorescent α-Factor Analogue Exhibits Multiple Steps on Binding to Its G Protein Coupled Receptor in Yeast. Biochemistry. 2004;43(42):13564–13578. doi:10.1021/bi0494018.

[6] Ventura AC, Bush A, Vasen G, Goldín MA, Burkinshaw B, Bhattacharjee N, et al. Utilization of extracellular information before ligand-receptor binding reaches equilibrium expands and shifts the input dynamic range. Proceedings of the National Academy of Sciences. 2014;111(37):E3860–E3869. doi:10.1073/pnas.1322761111.
