# Supplementary material for: Information bounds on the accuracy of cell polarization
Source: PLoS One. 2025 Sep 30;20(9):e0333522. doi: 10.1371/journal.pone.0333522 (PMC12483228; doi:10.1371/journal.pone.0333522)
Supplement: S1 Text — (PDF) [file pone.0333522.s007.pdf]

S1 Text. Derivation of expression for receptor measurement noise.

Under given biological conditions, what is the estimate of N, the measurement noise of a single receptor? As described in the Introduction, previous work has derived estimates of N using various methods. The noise expression contains two terms: the variance from diffusion and the variance from stochastic receptor-ligand binding. However, there are subtle differences depending on the derivation. Thus, we adopted a new approach based on the Poisson mixture distribution [1, 2, 3] to offer a new perspective and compare with previous work.

A single receptor on a patch of cell surface membrane measures the concentration of a ligand in its local neighborhood via binding to the ligand (Fig 1C). This measurement is corrupted by noise, which we wish to estimate. The binding process consists of two stochastic stages: 1) diffusion of ligand in/out of the local neighborhood of the receptor, and 2) binding/unbinding to receptor. We adopt the assumption [4] that the local neighborhood is well-mixed with the global solvent environment. We define the neighborhood as a sphere of radius s around the receptor.

Let A_b represent the number of binding events, and A_u represent the number of unbinding events in a time interval T for a receptor. From chemical kinetics, $A_b = k_a cT(1-p)$ where k_a is the association rate constant, c is the concentration of ligand, and p is the receptor occupancy. The $(1-p)$ term represents unbound receptor that are capable of binding ligand. At steady-state, $A_b = k_a c(1-p) = k_d p = A_u$, where k_d is the dissociation rate constant. Hence c can be written in terms of A_b and A_u.

Using the rule of error propagation [5], we express the fractional variance of c as: $\left(\frac{\delta c}{c}\right)^2 = \frac{1}{c^2}\left(\frac{\partial c}{dA_b}\right)^2 \mathrm{Var}[A_b] + \frac{1}{c^2}\left(\frac{\partial c}{dA_u}\right)^2 \mathrm{Var}[A_u]$. We know that $\frac{\partial c}{\partial A_b} = \frac{1}{k_a T(1-p)} = \frac{\partial c}{\partial A_u}$ so that $\left(\frac{\delta c}{c}\right)^2 = \frac{1}{(k_a cT(1-p))^2}\left(\mathrm{Var}[A_b] + \mathrm{Var}[A_u]\right)$.

Ligand unbinding is a Poisson process possessing an average of $k_d pT = k_a cT(1-p)$, and hence a variance of $\mathrm{Var}[A_u] = k_a cT(1-p)$. Estimating $\mathrm{Var}[A_b]$ is more complicated because binding depends on the local concentration of ligand in the receptor neighborhood which is subject to fluctuations from diffusion. We can think of binding as the result of two successive Poisson processes: diffusion followed by the binding event.

In a mixed Poisson distribution, the random variable is Poisson distributed while the rate parameter λ is also a random variable so that the probability density $f(x) = \int_\Theta f(x|\lambda)g(\lambda)d\lambda$ where $\lambda \in \Theta$. Furthermore for the mixed Poisson distribution, $\mathrm{Var}[X] = E[\lambda] + \mathrm{Var}[\lambda]$.

If we let c_l be the concentration of ligand in the local neighborhood of receptor and c be the surrounding concentration, then for the random variable A_b the Poisson parameter $\lambda = k_a c_l T(1-p)$. We have $E[\lambda] = k_a cT(1-p)$, and $\mathrm{Var}[\lambda] = (k_a T(1-p))^2 \mathrm{Var}[c_l]$ so that $\mathrm{Var}[A_b] = k_a cT(1-p) + (k_a T(1-p))^2 \mathrm{Var}[c_l]$.

The variable c_l depends on the diffusive flux into the local receptor neighborhood, f_i, and the diffusive flux out of the neighborhood, f_o. Once again

applying the rule of error propagation, we obtain $\mathrm{Var}[c_l] = \left(\frac{\partial c_l}{\partial f_i}\right)^2 \mathrm{Var}[f_i] + \left(\frac{\partial c_l}{\partial f_o}\right)^2 \mathrm{Var}[f_o]$. For a given receptor, the flux out from diffusion over a time span T from the receptor neighborhood of radius s is $f_o = 4\pi s D c_l T$ molecules/s.

At steady-state, the average flux in equals the average flux out. Because this is a Poisson process, the variance is the same as the mean, which is equal to the inward average flux and variance. We thus have $\mathrm{Var}[f_i] = \mathrm{Var}[f_o] = 4\pi s DcT(1-p)$ with the factor $(1-p)$ representing the unoccupied receptors since we are estimating binding variance. Because $\frac{\partial c_l}{\partial f_i} = \frac{1}{4\pi sDT(1-p)} = \frac{\partial c_l}{\partial f_o}$, $\mathrm{Var}[c_l] = \left(\frac{2c}{4\pi sDT(1-p)}\right)$. We can now write $\mathrm{Var}[A_b] = k_a cT(1-p) + \frac{2c(k_a T(1-p))^2}{4\pi sDT(1-p)}$.

Finally, putting it together,

$$\begin{aligned}\left(\frac{\delta c}{c}\right)^2 &= \left(\frac{1}{(k_a cT(1-p))^2}\right)\left(k_a cT(1-p) + \frac{2c(k_a T(1-p))^2}{4\pi sDT(1-p)} + k_a cT(1-p)\right)\\ &= \frac{2}{4\pi sDcT(1-p)} + \frac{2}{k_a cT(1-p)}.\end{aligned}$$

This expression is identical to that of Kaizu et al. [6] and differs from Bialek and Setayeshgar [7] by a factor of $2(1-p)$ in the diffusion term.

References

[1] Willmot G. Mixed Compound Poisson Distributions. ASTIN Bulletin: The Journal of the IAA. 1986;16(S1):S59–S79. doi:10.1017/S051503610001165X.

[2] Karlis D, Xekalaki E. Mixed Poisson Distributions. International Statistical Review / Revue Internationale de Statistique. 2005;73(1):35–58.

[3] Neyman J. On a New Class of "Contagious" Distributions, Applicable in Entomology and Bacteriology. The Annals of Mathematical Statistics. 1939;10(1):35–57. doi:10.1214/aoms/1177732245.

[4] ten Wolde PR, Becker NB, Ouldridge TE, Mugler A. Fundamental Limits to Cellular Sensing. J Stat Phys. 2016;162(5):1395–1424. doi:10.1007/s10955-015-1440-5.

[5] Taylor JR. An Introduction to Error Analysis: The Study of Uncertainties in Physical Measurements. 2nd ed. Sausalito, Calif: University Science Books; 1996.

[6] Kaizu K, de Ronde W, Paijmans J, Takahashi K, Tostevin F, ten Wolde P. The Berg-Purcell Limit Revisited. Biophys J. 2014;106(4):976–985. doi:10.1016/j.bpj.2013.12.030.

[7] Bialek W, Setayeshgar S. Physical limits to biochemical signaling. Proceedings of the National Academy of Sciences. 2005;102(29):10040–10045. doi:10.1073/pnas.0504321102.
