# Supplementary material for: Information bounds on the accuracy of cell polarization
Source: PLoS One. 2025 Sep 30;20(9):e0333522. doi: 10.1371/journal.pone.0333522 (PMC12483228; doi:10.1371/journal.pone.0333522)
Supplement: S2 Text — (PDF) [file pone.0333522.s008.pdf]

S2 Text. Equations for Coop, PF, and FA models.

The Coop and PF models are described in greater detail in Chou et al. [1, 2]. The FA model possesses a first-order filter in front of the PF model. In all three models, u is the input, a is the polarized species on the surface of the 2D disk ($r = 1$ μm), and b is the negative feedback species that is well-mixed in all of the compartments. $D = 0.001$ $\mu m^2/s$ is the surface diffusion coefficient.

Cooperative Model (Coop)

$$\frac{\partial a}{dt} = D\nabla_s^2 a + \frac{k_0}{1+(\beta u)^{-q}} - k_2 a - k_3 ba$$
$$\frac{db}{dt} = k_4 \hat{a} b$$

where $\hat{a} = \bar{a} - a_{ss}$ and $\bar{a} = \frac{\int_s ads}{\int_s ds}$. $k_0 = 10$, $k_2 = k_3 = k_4 = 1$, $\beta = 1$, $q = 1000$, $a_{ss} = 1$.

Positive Feedback Model (PF)

$$\frac{\partial a}{dt} = D\nabla_s^2 a + \frac{k_0}{1+(\beta u)^{-q}} + \frac{k_1}{1+(\gamma a)^{-h}} - k_2 a - k_3 ba - k_5 \hat{a}$$
$$\frac{db}{dt} = k_4 \hat{a} b$$

where $\hat{a} = \bar{a} - a_{ss}$ and $\bar{a} = \frac{\int_s ads}{\int_s ds}$. $k_0 = 1$, $k_1 = 10$, $k_2 = k_3 = k_4 = 1, k_5 = 10$, $\beta = 1$, $\gamma = \frac{1}{1+u^{-q}}$, $q = 100$, $h = 2$, $a_{ss} = 1$.

Filter-Amplifier Model (FA)

The FA model is composed of three equations in which the first equation is a first-order filter with time constant τ, while the second and third equations are the positive feedback (PF) model.

$$\frac{\partial f}{\partial t} = \frac{u - f}{\tau}$$
$$\frac{\partial a}{dt} = D\nabla_s^2 a + \frac{k_0}{1+(\beta f)^{-q}} + \frac{k_1}{1+(\gamma a)^{-h}} - k_2 a - k_3 ba - k_5 \hat{a}$$
$$\frac{db}{dt} = k_4 \hat{a} b$$

where $\tau = 10$, and the other parameters are specified above in the PF model.

References

[1] Chou CS, Nie Q, Yi TM. Modeling Robustness Tradeoffs in Yeast Cell Polarization Induced by Spatial Gradients. PLOS ONE. 2008;3(9):e3103. doi:10.1371/journal.pone.0003103.

[2] Chou CS, Bardwell L, Nie Q, Yi TM. Noise filtering tradeoffs in spatial gradient sensing and cell polarization response. BMC Systems Biology. 2011;5(1):196. doi:10.1186/1752-0509-5-196.
